# Prevalence of physical and verbal aggressive behaviours and associated factors among older adults in long-term care facilities

**DOI:** 10.1186/1471-2318-5-13

**Published:** 2005-11-10

**Authors:** Philippe Voyer, René Verreault, Ginette M Azizah, Johanne Desrosiers, Nathalie Champoux, Annick Bédard

**Affiliations:** 1 Faculty of nursing, Laval University, Quebec City, Canada; 2 Faculty of Medicine, Laval University, Quebec City, Canada; 3 Faculty of Medicine, University of Sherbrooke, Sherbrooke, Canada; 4 Faculty of Medicine, University of Montréal, Montreal, Canada; 5 School of Psychology, Laval University, Quebec City, Canada

## Abstract

**Background:**

Verbal and physical aggressive behaviours are among the most disturbing and distressing behaviours displayed by older patients in long-term care facilities. Aggressive behaviour (AB) is often the reason for using physical or chemical restraints with nursing home residents and is a major concern for caregivers. AB is associated with increased health care costs due to staff turnover and absenteeism.

**Methods:**

The goals of this secondary analysis of a cross-sectional study are to determine the prevalence of verbal and physical aggressive behaviours and to identify associated factors among older adults in long-term care facilities in the Quebec City area (n = 2 332).

**Results:**

The same percentage of older adults displayed physical aggressive behaviour (21.2%) or verbal aggressive behaviour (21.5%), whereas 11.2% displayed both types of aggressive behaviour. Factors associated with aggressive behaviour (both verbal and physical) were male gender, neuroleptic drug use, mild and severe cognitive impairment, insomnia, psychological distress, and physical restraints. Factors associated with physical aggressive behaviour were older age, male gender, neuroleptic drug use, mild or severe cognitive impairment, insomnia and psychological distress. Finally, factors associated with verbal aggressive behaviour were benzodiazepine and neuroleptic drug use, functional dependency, mild or severe cognitive impairment and insomnia.

**Conclusion:**

Cognitive impairment severity is the most significant predisposing factor for aggressive behaviour among older adults in long-term care facilities in the Quebec City area. Physical and chemical restraints were also significantly associated with AB. Based on these results, we suggest that caregivers should provide care to older adults with AB using approaches such as the progressively lowered stress threshold model and reactance theory which stress the importance of paying attention to the severity of cognitive impairment and avoiding the use of chemical or physical restraints.

## Background

Among the entire spectrum of behavioural and psychological symptoms of dementia, aggressive behaviour (AB) is the most disturbing and distressing behaviour displayed by older patients in long-term care facilities. According to Patel and Hope [1], AB refers to an overt act, which is not accidental, involving the delivery of noxious stimuli to (but not necessarily aimed at) an object or towards the self or others. Choux and colleagues [2] further specified that the AB may be verbal or physical behaviour that harms or threatens another person. Physical aggression includes hitting, kicking, scratching, pushing, biting, punching, grabbing, throwing objects, pinching, cutting, and stabbing. Verbal aggression is typically considered as insulting, obscene or profane language or sexual advances.

The purpose of this study was to describe the phenomenon of AB among older patients in long-term care facilities.

### Prevalence of AB among older patients in long-term care facilities

Prevalence of aggressive behaviour among residents in long-term care facilities varies widely from 7% to 91% [3-7]. According to a recent literature review [8], it was estimated that on average 24% of cognitively impaired residents are agitated or aggressive. Based on our own review of studies published between 1999 and 2001, 24 to 95% of long term care residents display AB, 10 to 95% exhibit physical aggressive behaviour and 10 to 91%, verbal aggressive behaviour [9,10]. In brief, prevalence rates of AB, although varying widely, may be very high.

### Consequences of aggressive behaviour

Aggressive behaviour affects mainly older patients themselves and their informal and formal caregivers. It can also lead to increased health care costs. Older patients displaying ABs and other disruptive behaviours are more likely to receive psychotropic drugs and to be physically restrained [7]. The negative consequences of both interventions are well known and include worsening of cognitive impairment and reduced physical strength, endurance and flexibility [11]. AB is also associated with depression and loss of functional independence [12].

Family members and friends are affected by ABs in long-term care facilities [13,14]. They can be embarrassed by these behaviours and eventually reduce the frequency of their visits.

Health care providers' stress is augmented by AB displayed by long-term care residents [7,15]. ABs are more likely to occur during the activities of daily living (ADL) [16], more specifically when the older patients are mobilized, transferred, dressed, fed, bathed and groomed. AB compromises the delivery of care and can lead to psychological distress, emotional exhaustion, depression and occupational injuries among nursing staff [2,17,18]. Finally, increased health care costs arising from staff turnover and absenteeism are also associated with the prevalence of AB among older patients in long term care facilities [16].

### Factors associated with aggressive behaviour

Individual and environmental factors have been associated with AB among older adults. Among the individual factors, being of male gender and among the younger segment of the older adult population (65 to 70 years old) are the most consistent demographic characteristics associated with AB [19,20]. Other individual factors include dementia (cortical and subcortical) and delirium, especially its hyperactive form [4,5,9,21]. Among older patients with dementia, severity of cognitive impairment has been linked to AB. Older patients with severe cognitive impairment are associated with more frequent AB episodes [19]. Psychiatric diseases (depression, mania, schizophrenia, anxiety and post traumatic stress disorders) and some specific symptoms (delusions, illusions, hallucinations) have also been linked to AB [5,19]. Personality traits, especially premorbid aggressive personality traits and aggression, are also risk factors for assaultive behaviour [2,6]. Researchers underline the potential role of pain, discomfort, sensory deficiencies (vision, hearing) and unmet basic needs (nutrition, hydration, exercise, sleep, etc) that could also lead to AB [19,21,22].

Both social and physical environmental factors, such as social visitation and physical restraint, may trigger an outburst of AB and, as has previously been mentioned, ABs occur more frequently during ADL provided by nursing staff. Researchers have hypothesized that older patients react in this way because their personal space or privacy is perceived as violated and they feel threatened [2,3,23]. Thus, AB would appear to be a defensive reaction. This stresses the importance of both the personal communication skills and knowledge and understanding of nursing staff providing care to older patients in institutions.

Environmental factors may also lead to ABs when they cause pain (e.g. drawing blood samples), discomfort or frustration (e.g. locked doors) or when they are interpreted as threatening. Similarly associated factors include noise, uncomfortable temperature, inadequate lighting and moving older patients to unfamiliar places [5,19].

To summarize, numerous factors have been associated with AB and since no one study has addressed all of them, it is difficult to determine those that are more important than others. This lack of knowledge concerning AB in long-term care facilities raises four important issues. First, studies on AB in long-term care facilities have generally been conducted on a small sized sample, some were case studies and few details are usually provided about methods for selecting participants [2,10,16,20,24,25], i.e. many studies appear to be vulnerable to selection bias. Second, many have examined less than four of the risk factors for AB [10,23-27], although there are obviously some exceptions to this observation [6,20]. Third, among those studies reporting prevalence rates for AB, few have described in detail the frequencies of specific ABs displayed in long-term care facilities [6,28]. Fourth, results of recent studies that have separated AB into verbal and physical behaviours suggest that factors associated with these two behaviour types may be different [25,27,28]. Based on their results, we are of the opinion that there is a sound basis for dividing AB into two different categories, namely verbal and physical AB.

### Objectives of the study

The main objective of this study was to describe the phenomenon of AB among older patients in long-term care facilities. Specific objectives were: 1 – to determine the prevalence of verbal and physical AB and the frequencies for each behaviour, and 2 – to identify the factors associated with a) both verbal and physical aggressive behaviours (BAB), b) physical aggressive behaviour (PAB) and, c) verbal aggressive behaviour (VAB).

## Methods

This is a secondary analysis of a cross-sectional study involving 2 633 older adults that was originally carried out to identify factors associated with the use of physical restraints in all 28 long-term care facilities in the Quebec City area.

In the province of Quebec, public long-term care facilities generally house older people with significant physical and mental problems [29,30]. The admission process to these facilities is managed by a single central committee, which evaluates admission requests with respect to medical diagnoses, severity of the loss of autonomy, and extent of the health care needs [31].

### Sample

In the original study, individuals were eligible if they were aged 65 or over and living in long-term care facilities, notably nursing homes, in the Quebec City area. Residents in six long-term care units within a large-scale psychiatric institution (n = 301) were excluded from this analysis (n = 2 332), as most of these residents had a life-long history of a psychiatric illness, usually used more psychotropic drugs and displayed AB in a context quite different from that of the average older patient in a long-term care facility.

### Data Collection

Original data were collected from two sources as follows: first, structured simultaneous interviews with two nurses who were familiar with the residents in their health-care unit, and second, a systematic review of the medical files by research assistants. This strategy aimed at minimizing the risk that research assistants would influence the course of the interviews with nurses (information bias). Individual variables included in this study are: age, sex, length of stay, functional autonomy, cognitive status, psychological distress, isolation and withdrawal behaviour, sleep disturbance. Environmental variables are: social visitation, physical restraint and neuroleptic and benzodiazepine drugs.

#### Variables measured during the interviews with nurses

##### Dependant variable: Aggressive behaviour

Types of AB were evaluated using the validated French version of the Cohen Mansfield Agitation Inventory (CMAI) [32,33], an instrument widely used in the field of psychogeriatrics. This instrument measures 29 disruptive behaviours classified into four groups: 1) aggressive physical behaviour, 2) non-aggressive physical behaviour, 3) aggressive verbal behaviour, and 4) non-aggressive verbal behaviour. These behaviour types are rated on the basis of observations gathered during the two weeks preceding data collection. In this analysis, we focused solely on two dimensions of the scale, namely, aggressive physical behaviour and aggressive verbal behaviour. If participants showed at least one instance of aggressive physical behaviour, they were classified as displaying said behaviour. Likewise, the older person who evinced aggressive verbal behaviour was classified as displaying that behaviour. In both cases, if the older persons showed no aggressive behaviour during the 2 weeks preceding the interview, they were classified as not displaying AB. Psychometric properties of the French adaptation of the CMAI have been previously studied and qualified as good: interrater reliability (r = 0.72; p < 0.05), temporal stability (r = 0.72; p < 0.05), internal consistency (Cronbach's alpha varying between 0.75 and 0.77; p < 0.05), concomitant reliability (r = 0.74; p < 0.05) and construct validity [34].

##### Independent variables

The MOSES (Multidimensional Observation Scale for Elderly Subjects) is an instrument that provides an overall evaluation of older adults. It consists of 40 closed questions regarding quantified observations carried out in the week preceding the data collection in five domains: (1) functional autonomy; (2) cognitive status; (3) psychological distress; (4) disruptive behaviour; (5) isolation and withdrawal behaviour. In this study, four domains (1, 2, 3 and 5) were used. We did not use the disruptive behaviour subscale because we preferred to use a better known scale for this variable (the CMAI, see above), since its extensive use offers excellent comparability. The MOSES was developed and validated with a sample of 2,391 patients in long-term care, aged 65 and over [35]. The MOSES scale has an internal consistency of 0.80 (p < 0.05). The internal consistencies of each domain of the scale are also satisfactory: functional autonomy 0,81, cognitive status 0,86, psychological distress 0,79, disruptive behaviours 0,78, isolation and withdrawal behaviour 0,77. Correlations with the Zung Depression, Robertson Short Mental Status, Kingston Dementia and the Physical and Mental Impairment-of-function Evaluation scales have confirmed the validity of the MOSES scale [35].

All of the MOSES variables were categorized by two clinician-researchers (PV, RV) according to two criteria. First, we looked at their clinical relevance and meaning. We wanted to ensure that any clinician could easily distinguish the categories created. Second, we looked at the mean, median and variance of data for each variable to be categorized. We wanted to ensure that the created categories would include a similar number of subjects or a sufficient number of subjects in each category. For instance, autonomous or semi-autonomous residents have to be easily distinguished from totally dependent residents by any health care provider.

*(1) Functional autonomy *is a composite variable integrating six items from the MOSES scale: dressing, bathing, aesthetic care, use of toilets, physical mobility, and getting in and out of bed. Each item was assigned one of four ratings, from 1 (entirely independent) to 4 (entirely dependent). For each resident, the ratings for the six items were added up to constitute a functional autonomy score varying between 6 and 24. For the study analyses, the total score was dichotomised in autonomous/semi-autonomous (rating of 6 to 20), and dependent (rating of 21 to 24).

*(2) Cognitive status *is a composite variable based on seven items from the MOSES scale: understanding communication, talking, recognizing personnel, perception of place, perception of time, memory of recent events, and memory of important past events. Each item was assigned one of four ratings, from 1 (no impairment) to 4 (severe impairment), and summed together for a total score varying from 7 to 28. For analysis, the score was divided into three categories: a) no cognitive impairment (rating of 7), b) mild-moderate cognitive impairment (rating between 8 and 21), c) severe cognitive impairment (rating between 22 and 28).

*(3) Psychological distress *(i.e. the combination of symptoms of depression and anxiety) was assessed by the following seven MOSES items: looking sad and depressed, reporting sadness and depression, sounding sad and depressed, looking worried and anxious, reporting worry and anxiety, crying, and pessimism about the future. Each item was rated (1 = never; 2 = sometimes; 3 = often; 4 = always). Participants were identified as psychologically distressed if they either often or always displayed at least one symptom of psychological distress during the previous week.

*(5) Isolation and withdrawal behaviour *were assessed usingeight items from the MOSES scale: prefers solitude, initiates social contacts, responds to social contacts, maintains friendships with other residents, shows interest in daily and external events, keeps busy, and helps other residents. Each of these items was rated from 1 (socially active) to 4 (socially isolated) for a total score varying from 8 to 32 for each participant. This variable was dichotomised: *No*, if the older person was socially active or slightly active (rating from 8 to 23) and *Yes*, if the older person was socially isolated or slightly isolated (rating from 24 to 32). Following variables were not measured by the MOSES.

###### Social visitation

The number of hours of visits by family and friends received by older adults on a monthly basis was calculated by averaging the number of hours of visits over the previous 12 months. This scale has been developed by our team.

###### Use of physical restraints during the 24 hours preceding the interview

Physical restraints include ties, straps or belts (which can be tied to the legs, ankles, arms or waist), jackets, gloves, geriatric chairs equipped with security tables, or other devices designed to limit the mobility of the older person and over which he or she has no control. Bedrails, half doors and locked doors forming a barrier or obstacle to keep the older person in a given area, were not considered as physical restraints. This scale has very good validity and reliability [36].

*Sleep disturbance *was determined by the presence of four symptoms of sleeping problems during the previous week. Two nurses were asked to use a Likert-type scale (1 = never to 4 = always) to rate whether the subject a) had trouble falling asleep, b) woke up and had trouble falling back to sleep during the night, c) woke up too early in the morning, and d) did not appear rested in the morning. According to DSM-IV-R [37], a subject is diagnosed with sleep disturbance if he or she displays difficulty initiating or maintaining sleep, or displays non-restorative sleep and it causes significant distress or impairment in daytime functioning. For the study, we classified participants as having a sleep disturbance if there was evidence of symptom (d) (either often or always) and one of the remaining symptoms (a, b, c). In other words, subjects were considered to have sleep disturbance if they had one of these combinations: (a + d), or (b + d), or (c + d). This scale was developed by our team for this study.

#### Variables measured by review of medical files

A structured questionnaire was used to collect information on: (1) *socio-demographic characteristics of participants*: *age, gender, and length of stay in the care unit*, (2) use of *benzodiazepines and neuroleptics. *To be considered consumers of benzodiazepine, participants had to have a regular prescription for or have consumed an as-needed (PRN) dose of this drug during the previous week. The identical procedure was followed for neuroleptics. The medication was coded according to the Anatomic, Therapeutic, Chemical (ATC) classification system [38]. Benzodiazepines which includes the short-acting and long-acting forms, are coded NO5BA in this system. Neuroleptics, both conventional and atypical, are coded NO5A.

The protocol of this study was approved by the Laval University Research Ethics Board.

### Statistical analysis

Characteristics of the participants and ABs (verbal, physical and both) were described by frequencies and percentages. Bivariate analyses to determine crude odds ratios were used to evaluate the degree of association between independent variables and ABs (verbal, physical and both). Statistically significant variables in bivariate analyses were then assessed for multicolinearity according to the method outlined by Besley and colleagues [39]. The isolating and withdrawal behaviours and the functional autonomy variables correlated strongly with cognitive impairment and were discarded in the final model. Logistic regression analysis was used to examine the contribution of each independent variable to ABs (verbal, physical and both). All analyses were carried out using the Statistical Analysis System (SAS) software, version 8.0.

## Results

Among the 2 332 residents who were included in this analysis, 494 (21.2%) displayed physical aggressive behaviour (PAB), 497 (21.5%) verbal aggressive behaviour (VAB) and 258 patients (11.2%) displayed both behaviours (BAB) (see Tables 1, 2, 3). Among all PAB measured in this study, hitting, pushing and kicking are the most common (Table 2). Among VAB, verbal aggression or insults were the most frequent (Table 3).

**Table 1:** Factors associated with both forms of aggressive behaviour (verbal and physical).

Characteristic	Total	Both forms of Aggressive behaviour		Bivariate analyses	Regression analyses
	N: 2309 (23 miss.)	Yes n:258 (11.2%)	No n: 2051 (88.8%)	Crude odds ratio (95% confidence interval)	Adjusted odds ratio (95% confidence interval)
**Individual factors**					
**Age **(years)					
≥ 85 ‡	1063 (45.6%)	110 (42.6%)	946 (46.1%)	1.00	1.00
75 to 84	893 (38.3%)	118 (45.7%)	764 (37.3%)	1.33* (1.01–1.75)	1.22 (0.91–1.63)
65 to 74	376 (16.1%)	30 (11.6%)	341 (16.6%)	0.76 (0.50–1.15)	0.61 (0.39–0.96)
**Gender**					
Female ‡	1759 (75.4%)	165 (63.9%)	1579 (77.0%)	1.00	1.00
Male	573 (24.6%)	93 (36.1%)	472 (23.0%)	1.89* (1.43–2.48)	2.13* (1.58–2.86)
**Length of stay in LTCC (years)**					
0 to 2 ‡	1093 (46.9%)	124 (48.1%)	951 (46.34%)	1.00	
3 to 4	432 (18.5%)	53 (20.5%)	375 (18.3%)	1.08 (0.77–1.53)	
≥ 5	807 (34.6%)	81 (31.4%)	725 (35.4%)	0.86 (0.64–1.15)	
**Functional autonomy**					
Autonomous / semi-autonomous‡	1207 (51.9%)	122 (47.3%)	1076 (52.5%)	1.00	
Dependent	1117 (48.1%)	136 (52.7%)	974 (47.5%)	1.23 (0.95–1.60)	
**Cognitive status**					
No impairment ‡	471 (20.3%)	15 (5.8%)	454 (22.2%)	1.00	1.00
Mild-moderate impairment	847 (36.5%)	91 (35.3%)	749 (36.5%)	3.69* (2.11–6.44)	2.87* (1.62–5.09)
Severe impairment	1006 (43.3%)	152 (58.9%)	847 (41.3%)	5.44* (3.16–9.37)	3.77* (2.12–6.72)
**Withdrawal behaviour**					
No ‡	1219 (52.5%)	98 (37.9%)	1111 (54.2%)	1.00	
Yes	1105 (47.6%)	160 (62.0%)	939 (45.8%)	1.93* (1.48–2.52)	
**Sleep disturbance**					
No ‡	2180 (93.8%)	230 (89.1%)	1936 (94.4%)	1.00	1.00
Yes	144 (6.2%)	28 (10.8%)	114 (5.6%)	2.07* (1.34–3.20)	1.95* (1.23–3.08)
**Psychological distress**					
No ‡	1838 (78.8%)	187 (72.5%)	1631 (79.5%)	1.00	1.00
Yes	494 (21.2%)	71 (27.5%)	420 (20.5%)	1.47* (1.10–1.98)	1.36* (1.00–1.85)
**Environmental factors**					
**Use of benzodiazepine drugs**					
No ‡	1344 (57.6%)	136 (52.7%)	1195 (58.3%)	1.00	
Yes	988 (42.4%)	122 (47.3%)	856 (41.7%)	1.25 (0.97–1.62)	
**Use of neuroleptic drugs**					
No ‡	1683 (72.2%)	138 (53.5%)	1525(74.35%)	1.00	1.00
Yes	649 (27.8%)	120 (46.5%)	526 (25.6%)	2.52* (1.94–3.28)	2.12* (1.61–2.81)
**Social visitation.**					
0 to 3 hours ‡	777 (33.6%)	99 (38.4%)	671 (32.9%)	1.00	1.00
4 to 15 hours	736 (31.8%)	84 (32.6%)	649 (31.8%)	0.89 (0.65–1.21)	0.94 (0.68–1.29)
≥ 16 hours	803 (34.7%)	75 (29.1%)	722 (35.4%)	0.71* (0.52–0.98)	0.90 (0.64–1.26)
**Use of physical restraints**					
No ‡	1576 (67.6%)	137 (53.1%)	1421 (69.3%)	1.00	1.00
Yes	756 (32.4%)	121 (46.9%)	630 (30.7%)	1.99* (1.53–2.59)	1.43* (1.07–1.92)

* Statistically significant p < 0.05

p‡ Reference Category

miss. = missing

**Table 2:** Types and frequencies of physical aggressive behaviours.

Physical aggressive behaviours	Frequency (percent %) n = 2332 (22 miss.)
**Hitting**	
Never	2022 (87.5%)
Rarely	105 (4.5%)
Sometimes	79 (3.4%)
Often	58 (2.5%)
Always	46 (2%)
**Pushing**	
Never	2120 (91.8%)
Rarely	53 (2.3%)
Sometimes	55 (2.4%)
Often	36 (1.5%)
Always	46 (2%)
**Kicking**	
Never	2157 (93.4%)
Rarely	52 (2.2%)
Sometimes	44 (1.9%)
Often	28 (1.2%)
Always	29 (1.3%)
**Scratching**	
Never	2216 (95.9%)
Rarely	32 (1.4%)
Sometimes	27 (1.2%)
Often	15 (0.6%)
Always	20 (0.9%)
**Spitting**	
Never	2235 (96.8%)
Rarely	23 (1%)
Sometimes	15 (0.6%)
Often	7 (0.3%)
Always	30 (1.3%)
**Throwing things**	
Never	2236 (96.8%)
Rarely	36 (1.5%)
Sometimes	20 (0.9%)
Often	8 (0.3%)
Always	10 (0.4%)
**Biting**	
Never	2270 (98.3%)
Rarely	20 (0.9%)
Sometimes	7 (0.3%)
Often	5 (0.2%)
Always	8 (0.3%)
**Hurting self**	
Never	2292 (99.2%)
Rarely	8 (0.3%)
Sometimes	3 (0.1%)
Often	3 (0.1%)
Always	4 (0.2%)
**Intentional falling**	
Never	2295 (99.3%)
Rarely	9 (0.4%)
Sometimes	3 (0.1%)
Often	1 (0.04%)
Always	2 (0.1%)

**Table 3:** Types and frequencies of verbal aggressive behaviours

Verbal aggressive behaviours	Frequency (%) n = 2332 (12 miss.)
**Verbal aggression or insult**	
Never	1902 (81.9%)
Rarely	141 (6.1%)
Sometimes	109 (4.7%)
Often	86 (3.7%)
Always	82 (3.5%)
**Verbal threat**	
Never	2072 (89.3%)
Rarely	76 (3.3%)
Sometimes	76 (3.3%)
Often	48 (2.1%)
Always	48 (2.1%)
**Verbal sexual advances**	
Never	2303 (99.3%)
Rarely	8 (0.3%)
Sometimes	5 (0.2%)
Often	4 (0.2%)
Always	0 (0%)

miss. = missing

In multivariate analyses, the individual factors significantly associated with BAB are: male gender (OR = 2.13), mild-moderate and severe cognitive impairment (respectively OR = 2.87 and 3.77), sleep disturbance (OR = 1.95), and psychological distress (OR = 1.36). Environmental factors associated with BAB are: neuroleptic drug use (OR = 2.12) and physical restraints (OR = 1.43) (Table 1).

About one fifth (21.2%) of older adults in long-term care displayed PAB. In the multivariate analysis (Table 4), individual factors associated with PAB are as follows: aged over 74 years (OR = 0.69), male gender (OR = 2.04), mild-moderate or severe cognitive impairment (respectively OR = 2.59 and 8.26), sleep disturbance (OR = 2.03) and psychological distress (OR = 1.31). Neuroleptic drug use (OR = 1.74) is the only environmental factor associated with PAB.

**Table 4:** Factors associated with physical aggressive behaviours.

Characteristic	Aggressive physical behaviours		Bivariate analyses	Regression analyses
	Yes n: 494 (21.2%)	No n: 1816 (77.8%)	Crude odds ratio (95% confidence interval)	Adjusted odds ratio (95% confidence interval)
**Individual factors**				
**Age **(years)				
≥ 85 ‡	228 (46.2%)	829 (45.6%)	1.00	1.00
75 to 84	203 (41.1%)	679 (37.4%)	1.09 (0.88–1.35)	1.06 (0.83–1.34)
65 to 74	63 (12.8%)	308 (16.9%)	0.74 (0.55–1.01)	0.69* (0.49–0.98)
**Gender**				
Female ‡	339 (68.6%)	1406 (77.4%)	1.00	1.00
Male	155 (31.4%)	410 (22.6%)	1.57* (1.26–1.95)	2.04* (1.59–2.62)
**Length of stay in LTCC **(years)				
0 to 2 ‡	225 (45.6%)	851 (46.9%)	1.00	
3 to 4	101 (20.5%)	327 (18.0%)	1.17 (0.89–1.53)	
≥ 5	168 (34.0%)	638 (35.1%)	0.99 (0.80–1.25)	
**Functional autonomy**				
Autonomous / semi-autonomous‡	187 (37.8%)	1012 (55.7%)	1.00	1.00
Dependent	307 (62.12%)	803 (44.3%)	2.07* (1.69–2.54)	0.79 (0.60–1.05)
**Cognitive status**				
No impairment ‡	23 (4.7%)	446 (24.6%)	1.00	1.00
Mild-moderate impairment	119 (24.1%)	722 (39.9%)	3.20* (2.02–5.08)	2.59* (1.61–4.17)
Severe impairment	352 (71.3%)	647 (35.7%)	10.57* (6.82–16.39)	8.26* (5.13–13.30)
**Withdrawal behaviour**				
No ‡	150 (30.4%)	1060 (58.4%)	1.00	
Yes	344 (69.9%)	755 (41.6%)	3.22* (2.60–3.99)	
**Sleep disturbance**				
No ‡	446 (90.3%)	1721 (94.8%)	1.00	1.00
Yes	48 (9.7%)	94 (5.2%)	1.97* (1.37–2.83)	2.03* (1.35–3.04)
**Psychological distress**				
No ‡	367 (74.3%)	1452 (79.9%)	1.00	1.00
Yes	127 (25.7%)	364 (20.0%)	1.38* (1.09–1.74)	1.31* (1.02–1.69)
**Environmental factors**				
**Use of benzodiazepine drugs**				
No ‡	286 (57.9%)	1046 (57.6%)	1.00	
Yes	208 (42.1%)	770 (42.4%)	0.99 (0.81–1.21)	
**Use of neuroleptic drugs**				
No ‡	285 (57.7%)	1379 (75.9%)	1.00	1.00
Yes	209 (42.3%)	437 (24.1%)	2.32* (1.88–2.85)	1.74* (1.38–2.19)
**Social visitation.**				
0 to 3 hours ‡	198 (40.2%)	572 (31.6%)	1.00	1.00
4 to 15 hours	159 (32.3%)	574 (31.7%)	0.81 (0.64–1.03)	0.94 (0.68–1.29)
≥ 16 hours	136 (27.6%)	662 (36.6%)	0.60* (0.47–0.77)	0.90 (0.64–1.26)
**Use of physical restraints**				
No ‡	241 (48.8%)	1318 (72.6%)	1.00	1.00
Yes	253 (51.2%)	498 (27.4%)	2.78* (2.27–3.41)	1.79 (1.37–2.33)

* Statistically significant p < 0.05

‡ Reference Category

miss. = missing

One fifth of the subjects under study (21.5%) displayed VAB. Table 5 shows the individual factors linked to VAB to be: male gender (OR = 1.64), functional dependency (OR = 0.78), mild-moderate or severe cognitive impairment (respectively OR = 1.85 and 1.48), and sleep disturbance (OR = 1.76). Environmental factors for VAB are benzodiazepine and neuroleptic drug use (respectively OR = 1.28 and 1.62). A summary of the factors associated with BAB, PAB, and VAB in the study is provided in Table 6.

**Table 5:** Factors associated with verbal aggressive behaviours.

Characteristic	Aggressive verbal behaviours		Bivariate analyses	Regression analyses
	Yes n: 497 (21.5%)	No n: 1823 (78.9%)	Crude odds ratio (95% confidence interval)	Adjusted odds ratio (95% confidence interval)
**Individual factors**				
**Age **(years)				
≥ 85 ‡	187 (37.6%)	870 (47.7%)	1.00	1.00
75 to 84	220 (44.3%)	668 (36.6%)	1.53* (1.23–1.91)	1.40 (1.12–1.76)
65 to 74	90 (18.1%)	285 (15.6%)	1.47* (1.11–1.95)	1.18 (0.87–1.60)
**Gender**				
Female ‡	329 (66.2%)	1422 (78.0%)	1.00	1.00
Male	168 (33.8%)	401 (22.0%)	1.81* (1.46–2.25)	1.64* (1.30–2.06)
**Length of stay in LTCC **(years)				
0 to 2 ‡	237 (47.7%)	845 (46.4%)	1.00	
3 to 4	96 (19.3%)	336 (18.4%)	1.02 (0.78–1.33)	
≥ 5	164 (33.0%)	642 (35.2%)	0.91 (0.73–1.14)	
**Functional autonomy**				
Autonomous / semi-autonomous‡	282 (56.8%)	923 (50.7%)	1.00	1.00
Dependent	214 (43.1%)	899 (49.3%)	0.78* (0.64–0.95)	0.78* (0.62–0.99)
**Cognitive status**				
No impairment ‡	76 (15.3%)	395 (21.7%)	1.00	1.00
Mild-moderate impairment	221 (44.5%)	624 (34.3%)	1.82* (1.37–2.43)	1.85* (1.37–2.50)
Severe impairment	199 (40.1%)	803 (44.1%)	1.27 (0.95–1.70)	1.48* (1.06–2.07)
**Withdrawal behaviour**				
No ‡	265 (53.4%)	951 (52.2%)	1.00	
Yes	231 (46.6%)	871 (47.8%)	0.95 (0.78–1.16)	
**Sleep disturbance**				
No ‡	449 (90.5%)	1725 (94.7%)	1.00	1.00
Yes	47 (9.5%)	97 (5.3%)	1.86* (1.29–2.68)	1.76* (1.21–2.56)
**Psychological distress**				
No ‡	373 (75.1%)	1454 (79.7%)	1.00	1.00
Yes	124 (24.9%)	369 (20.2%)	1.31* (1.04–1.65)	1.19 (0.93–1.51)
**Environmental factors**				
**Use of benzodiazepine drugs**				
No ‡	261 (52.5%)	1075 (58.9%))	1.00	1.00
Yes	236 (47.5%)	748 (41.0%)	1.30* (1.07–1.59)	1.28* (1.05–1.58)
**Use of neuroleptic drugs**				
No ‡	313 (62.9%)	1359 (74.5%)	1.00	1.00
Yes	184 (37.0%)	464 (25.4%)	1.72* (1.40–2.13)	1.62* (1.30–2.02)
**Social visitation.**				
0 to 3 hours ‡	167 (33.7%)	606 (33.4%)	1.00	
4 to 15 hours	176 (35.5%)	560 (30.9%)	1.15 (0.91–1.46)	
≥ 16 hours	153 (30.8%)	648 (35.7%)	0.86 (0.68–1.11)	
**Use of physical restraints**				
No ‡	325 (65.4%)	1242 (68.1%)	1.00	
Yes	172 (34.6%)	581 (31.9%)	0.13 (0.92–1.40)	

* Statistically significant p < 0.05

‡ Reference Category

miss. = missing

**Table 6:** Summary of factors associated with aggressive behaviours according to multivariate analysis.

**Associated risk factors**	**BAB**	**PAB**	**VAB**
**Individual factors**			
Age (years) 85 and over		+	
Male gender	+	+	+
Functionally independent			+
Mild-moderate or severe cognitive impairment	+	+	+
Sleep disturbance	+	+	+
Psychologically distressed	+	+	
**Environmental factors**			
Use of benzodiazepine drugs			+
Use of neuroleptic drugs	+	+	+
Use of physical restraints	+		

+ = statistically significant

## Discussion

### Prevalence of AB

The first goal of this study was to determine the prevalence of verbal and physical aggressive behaviour among older residents in long-term care settings in the Quebec City area. In this study, aggressive behaviour (either physical or verbal) was displayed by 21% of the older residents and 11.2% of them exhibited both forms. In other studies, Cohen-Mansfield et al. [3] reported a prevalence rate for AB of 8 to 91% in institutional settings, Lyketsos and colleagues [40] found a prevalence rate of 23.7% for aggressive/agitated behaviour (Neuropsychiatric Inventory) among community-dwelling seniors and long term care residents, and Schreiner [23] reported a prevalence rate of 45.4% of physical or verbal aggressive behaviour (CMAI; last two weeks) among 391 cognitively impaired long term care residents. Marx et al. [6] reported a lower rate of 32% for aggressive behaviour (physical and verbal) in long-term care facilities (CMAI; last two weeks) and lastly, Giancola et al. [4] reported prevalence rates of 14 to 21% for physical aggressive behaviour and 10–14% for verbal aggressive behaviour in two nursing homes. While our results are in general agreement with these reported prevalence rates there is, nevertheless, substantial discrepancy between prevalence rates among studies. This is probably due in part to their use of different measures of AB [23,40]. However we also cannot ignore the fact that some differences may be due to the varying quality of care provided in those long-term care facilities or to the inclusion criteria applied in the studies.

In our study, we found that 21% of older residents in long-term care settings displayed physical or verbal aggressive behaviour, confirming the significance of the phenomenon and the need to address it. Based on both their high frequency and their potentially distressing effect on both the resident and the caregivers, it appears that some specific behaviours, such as hitting and insults, deserve more attention from researchers. Such behaviour types have also been highlighted by researchers in previous studies [10,23].

Although there is a relatively high prevalence of ABs (21%), the majority are not displayed often. Indeed, less than 3% are displayed "often" or "always". We understand from these low frequency levels that, in general, older residents do not favour one behaviour over another when exhibiting AB. There is also the possibility that specific contexts of care giving are more conducive to aggressive behaviour. However we were not able to take this into account in our study. For instance, AB during bathing has previously been observed in half of older patients with dementia [41], an observation that has led researchers to target specific care-giving contexts to tackle the problem among older patients with dementia. Sloane et al. [42] tested two experimental bathing interventions compared to a control group leading to impressive results. When compared to the control group, aggressive behaviour in the person-centred shower group and in the towel-bath group declined significantly (53% and 60% respectively). This would appear a likely avenue for future research, to target specific contexts where AB is more likely to occur in order to develop intervention suited to those contexts. Another study [43] was successful in decreasing agitation among older residents in institutions by targeting many factors and contexts (see Clinical implications). These results suggest that a broad approach to nursing care is also of value in the prevention of AB in long-term care facilities and that specific interventions should be developed targeting those care activities at increased risk for AB.

Our second study goal was to identify the factors associated with BAB, PAB and VAB. Overall, they were quite similar. This is somewhat in line with the results of previous research studies, although they yielded opposite results [27,28]. Ryden et al. [27] found that among 116 residents, the use of psychotropic drugs, physical restraint and living on secured units were differently associated with VAB and PAB. In our study, use of benzodiazepine drugs was associated with VAB only and physical restraint only with BAB. It should be noted that Ryden's study focused exclusively on residents with frequent aggressive behaviours (9–10 aggressive behaviours a day, using the Ryden Aggression Scale-2), which is higher than the AB frequency in our study population. Using the CMAI, Schreiner [23] reported a higher prevalence of PAB among men than women (p = 0.05; n = 391), but did not observe such a difference for VAB, whereas we found men exhibited more PAB and VAB than did women. By examining patterns of co-occurrence of both ABs among residents (n = 240; 98% men; 32% had a psychiatric diagnosis), Souder et al [28] found PABs more likely to occur with non-aggressive physical behaviour, and unlikely to occur with verbally disruptive behaviours. These researchers did not look at other risk factors for AB. In our study, 11% of participants displayed BAB.

As can be concluded from these studies, much more research is needed in the field to determine whether the factors associated with BAB, PAB and VAB differ. Improved study comparability in the future requires research studies of comparable design and instruments. At present, differences in conclusions among studies could result from methodological differences, such as the instruments used, the Ryden Aggression Scale-2 [27], the CMAI [23], or the disruptive behaviour scale [28]. Nonetheless, certain results from our findings do deserve further attention. They are discussed below.

### AB and cognitive impairment

As shown in Tables 1, 4 and 5, AB was more likely to occur among older participants with mild-moderate or severe cognitive impairment than among those with no cognitive impairment. These findings are in agreement with those of Ryden et al. [27] and Marx et al. [6]. Menon et al. [26] also indicated that physical and verbal aggression increases with the severity of the cognitive impairment. According to Hall and O'Connor's literature review [19], the association between severity of cognitive impairment and aggressive behaviour has received strong support and may be caused by the communication deficiencies accompanying severe cognitive impairment.

This association between AB and cognitive impairment also provides support for the Progressively Lowered Stress Threshold (PLST) model [44,45], according to which, a person with dementia has a declining ability to adjust to environmental demands as the cognitive losses progress. Demands not adapted to the resident's stress threshold become stressors directed at the resident, leading to the development of anxiety symptoms. If the caregiver does nothing to reduce these stressors, the resident will then display agitation such as verbal or physical aggressive behaviours. This model is useful for explaining the association between severe cognitive impairment and aggression and as such, it warrants further attention from researchers and clinicians since it brings insight to our understanding of AB [45]. Said model has also been found to improve the quality of care provided by caregivers in the community [46,47] and therefore it would be important to also test its usefulness among older residents with severe cognitive impairment in long-term care facilities.

An interesting outcome of our study not in accordance with the PLST model is the non-linear association between cognitive impairment and VAB. According to the PLST, participants with severe cognitive impairment would have displayed more VAB than those with mild-moderate cognitive impairment. Our results in fact, showed that older residents with mild-moderate cognitive impairment were more likely to display VAB than those with severe cognitive impairment. Matteau et al. [25] explored the relationship between language deterioration and disruptive vocalization in demented residents (Alzheimer's, vascular or mixed type) living in nursing homes. They showed that those with language deficiencies were more likely to display frequent verbal behaviour in a large variety of distinct forms. Thus, disruptive vocalization could be a consequence of communicative difficulties [14,24,48]. Therefore, it is possible that among residents with severe cognitive impairment, their language limitations are so severe that they can no longer express their concerns through verbal agitation, including VAB. On the other hand, those residents with mild-moderate cognitive impairment, who possess residual verbal capacity, would be more prone to display their concerns via VAB, as was suggested by our results. As put forward by Hall and O'Connor [19], the association between communication impairment and VAB has implications for communication skill training for nursing staff.

### AB and psychotropic drug use

The use of neuroleptics was significantly associated with PAB, and both benzodiazepine and neuroleptics were linked to VAB. Other studies [7,10,14,20,40] have found AB and psychotropic drugs such as neuroleptics and benzodiazepines to be associated. Conventional (e.g. haloperidol, thioridazine, chlorpromazine) and atypical (e.g. risperidone, olanzapine, quetiapine) neuroleptics are frequently used in the treatment of agitation and AB among older residents. Three meta-analyses concluded that, despite their wide use, neuroleptics might reduce the frequency of disruptive behaviour by only 18% [49] to 26% in older patients with dementia [50]. Lonergan and colleagues [51] report that haloperidol is not more effective than a placebo in controlling agitation among the elderly suffering from dementia and was slightly more effective than a placebo for AB. In addition, these drugs are associated with frequent adverse effects such as extra-pyramidal symptoms, drowsiness and anticholinergic manifestations [52,53]. According to a one-year longitudinal study [54], researchers reported that change in disruptive behaviour occurs among nursing home residents regardless of the use of neuroleptic drugs, but that it occurs more frequently among those receiving neuroleptic medication. In fact, users of neuroleptics showed greater changes in both developing and resolving disruptive behaviour during the year than those not receiving the drugs. In short, given their limited effectiveness and the high risk of side effects, including permanent consequences such as tardive dyskinesia, the use of neuroleptic drugs should be a last resort

Benzodiazepines should also be used with caution among older residents. Liebson [9] reported that these drugs exacerbate cognitive deficits in cognitively impaired residents. Finally, we are not aware of any controlled clinical trial on the effectiveness of benzodiazepine for treating aggressive behaviour among older residents with dementia. It is worth mentioning that since sleep disturbance and psychological distress (both associated with AB) are indications for the use of benzodiazepine drugs, there are, undoubtedly, a certain percentage of participants who were taking these drugs to treat said symptoms and not AB. Nonetheless, future studies should be directed toward the development of new drugs and alternative treatments such as the towel bath [42] or staff training programs [55].

### AB and physical restraints

Physical restraints are sometimes used in an attempt to control aggressive or other risky behaviours. Their use results in loss of personal autonomy and self-esteem, and may lead to AB among residents. As found in a previous study [27], physical restraint (an environmental factor) was associated with AB among older residents. Tinetti et al. [56] reported that disruptive behaviour (which includes AB) was the reason most often cited for nursing staff's resorting to a physical restraint. However, physical restraint is not a solution for such behaviour [11]. Based on the reactance theory, we even suggest that physical restraint increases AB. Reactance theory, as proposed by Brehm, suggests that individuals pursue freedom and want control over their lives [57]. Any attempt to remove this sense of control or freedom from an individual will result in defensive behaviour. The removal of fundamental rights for a long period can lead to aggressive behaviour [58]. Therefore, one of the first interventions to be applied in the context of AB would be to reduce the use of physical restraints in long-term care settings.

### Clinical implications

Based on our results, we would like to suggest a preventive intervention program for AB inspired by the work of Inouye in the field of delirium [59]. Inouye et al. have been able to reduce the prevalence of delirium by implementing preventive interventions for every factor associated with delirium (e.g.: dehydration, malnutrition, sleep disturbance, hearing impairment, physical restraint, etc.). In the same way, it might be useful to adopt this approach for AB, since it too is associated with several factors. However a future study would need to test any such program to determine its relevance and effectiveness. According to our results, a multi-component intervention program would target the following five factors in order to reduce AB among older residents in long-term care facilities: cognitive impairment, sleep disturbance, psychological distress, benzodiazepine and neuroleptic drugs and physical restraints. Cognitive impairment, although it cannot be cured, can be alleviated somewhat through the use of an appropriate communication method. When the approach selected is suitable (validation therapy, reality orientation, reminiscence, etc.), it may reduce the occurrence of aggressive behaviours.

These multiple targets might discourage even the most well-intentioned caregivers and while it may appear that our recommendations are heavy artillery for treating AB with little real clinical application, a recent intervention study on agitation in long-term care [43] has been successful in targeting multiple risk factors for agitation, while remaining realistic in terms of clinical practice. We are of the opinion that this intervention, entitled BACE (Balancing Arousal Controls Excesses), appears promising, at least with regard to its approach to behavioural problems.

### Limits and contribution of the study

This study has some limitations. First, since it was cross-sectional in nature, the findings cannot be regarded as providing a cause-effect relationship. Second, aggressive behaviour, like all human behaviour, is influenced by individual (e.g. Parkinson's Disease, stroke) and environmental factors (e.g. staff's approach, environmental changes, quality of medical and nursing care), all of which could not be included in this study. Third, this study did not collect data on pain, a factor related to AB among long-term care residents. Lastly, this research is based on staff-reported data. The fact that the staff members in the institutions were very busy and had limited time available for the study may have affected the quality of the data collected. However, to reduce the impact of any potential bias, we interviewed two nurses simultaneously. Nevertheless, this work has two important strengths related to comprehensiveness: It should be noted in particular that our study population comprised all the residents in all the long-term care facilities in the Quebec City area (except for those in specialized psychiatric settings), and this resulted in a large sized sample. Finally, this study has made an important contribution by differentiating VAB and PAB when conducting analyses.

## Conclusion

Findings of the study suggest that overall, AB is associated with many individual factors (younger age, male gender, functional dependency, cognitive impairment, sleep disturbance, psychological distress) and environmental factors (benzodiazepine and narcoleptic drug use, physical restraints). Future prevention and treatment studies on AB are encouraged to pay attention to these factors and to be multi-dimensional in nature so as to better reflect our understanding of their association with AB. Reactance theory and the Progressively Lowered Stress Threshold model appear to us interesting frameworks that can improve nursing care in long-term care and reduce the prevalence and the burden of AB.

## List of Abbreviations Used

MOSES: Multidimensional Observation Scale for Elderly Subjects

PLST: Progressively Lowered Stress Threshold model

AB: aggressive behaviour

ABs: the different manifestations of aggressive behaviour

PAB: physical aggressive behaviour

VAB: verbal aggressive behaviour

## Competing interests

The author(s) declare that they have no competing interests.

## Authors' contributions

PV designed the secondary analysis study, supervised statistical analysis, outlined the first draft and edited subsequent drafts. RV participated in the design of the original study and edited the final draft. GA revised the literature review, wrote the first draft and edited subsequent drafts. JD, NC, AB edited first and subsequent drafts. All authors read and approved the final manuscript.

## Pre-publication history

The pre-publication history for this paper can be accessed here:


